# Translational Medicine and Implementation Science

**DOI:** 10.1590/1516-3180.2022.1416011123

**Published:** 2024-03-11

**Authors:** Protásio Lemos da Luz, Paulo Manuel Pêgo-Fernandes

**Affiliations:** I MD, PhD. Fellow American College of Cardiology, Senior Professor of Cardiology (Researcher), Faculty of Medicine, Universidade de São Paulo (USP), São Paulo (SP), Brazil.; II MD, PhD. Vice-director, Faculty of Medicine, Universidade de São Paulo (USP), São Paulo (SP), Brazil; Full Professor, Department of Cardiopneumology, Faculty of Medicine, Universidade de São Paulo (USP), São Paulo (SP), Brazil; Director of the Scientific Department, Associação Paulista de Medicina (APM), São Paulo (SP), Brazil.

In the past, numerous groundbreaking discoveries remained confined to basic sciences, taking decades to translate into diagnostic tools or treatments applicable to clinical practice.

A prime example is the association between cholesterol and atherosclerosis. Between 1908 and 1913, Russian researchers made the first observation of cholesterol-induced atherosclerosis in rabbits.^1^ The Framingham Heart Study,^2^ published in 1961, provided the first human evidence supporting this link. However, it was not until 1976 that the first statin drug was developed, beginning the modern era of atherosclerosis treatment.^3^ This significant delay, mirrored in numerous other instances, represents a loss of valuable knowledge and human lives.

Translational Medicine emerged to address this challenge, encompassing three primary areas: a) accelerating the transfer of knowledge from basic research to clinical application; b) elucidating the causal mechanisms and pathophysiology of clinical observations through interaction with basic science; and c) implementing basic knowledge and concepts derived from clinical and experimental research into the general population, a field known as Implementation Science.

Implementation science demands consideration of multiple factors.

## PERSONALIZED MEDICINE

Currently, medication prescriptions are based on research that has identified effective drug dosages. This approach fails to consider individual responses, focusing on the average rather than differentiating between responders and non-responders. Side effects are also reported in this manner. Randomized studies, on the contrary, exclude patients with comorbidities and only represent 6%–8% of the affected population, failing to reflect real-world scenarios. These limitations lead to errors and challenges in dose adjustment.

Pharmacogenetics offers a more precise understanding of patient responses to external agents, enabling personalized treatment strategies, such as preventing allergic reactions. In essence, understanding the human genome and bodily responses will pave the way for personalized treatments, taking into account individual reactions to contrast media, intolerance to external agents, salt sensitivity, and responses to antiplatelet and anticoagulant medications. While this is not yet standard practice, it is poised to become the norm in the near future.

## SOCIOECONOMIC INEQUALITIES INFLUENCE DISEASES

The Whitehall Study,^4^ conducted in the 1980s, demonstrated an association between lower job satisfaction and increased mortality. Subsequent research consistently shows that educational level, financial resources, and social status significantly impact disease prevalence and mortality rates.^5^ The underlying mechanisms extend beyond psychological factors. Individuals with higher socioeconomic status are generally more informed about health issues, have access to better medical facilities, and can afford healthcare expenses. This universal issue, deeply intertwined with economic and social development, manifests in disparate health outcomes.

## AGE AFFECTS THE COURSE OF ALL DISEASES

The global population is aging at an unprecedented rate. Comorbidities, such as cancer, cardiovascular diseases, rheumatic disorders, renal diseases, metabolic disorders, inflammatory diseases, urological disorders, respiratory diseases, neurological disorders (including dementia and Alzheimer’s), and psychiatric disorders, are highly prevalent among the older population. It is increasingly rare to find an older patient with only one disease, which underscores the need for multiple experts to collaborate and determine the most effective treatment approach in increasingly complex cases.^6,7^ Consistent with this idea, a meta-analysis concluded that teamwork positively correlates with clinical outcomes.^8^

## RISKS *VERSUS* BENEFITS OF MODERN TECHNOLOGIES

New technological advances in healthcare offer a multitude of benefits but also carry inherent risks. For instance, the ability to detect minimal thyroid, breast, and prostate lesions has led to unnecessary “preventive” interventions,^9^ potentially causing harm and anxiety to patients. Similarly, imaging techniques such as scintigraphy, coronary computed tomography angiography, and percutaneous interventions can be misused, overburdening healthcare systems, escalating costs, and causing distress in patients.

Countries like the United Kingdom and Canada have already implemented measures to curb excessive use of these technologies. In Brazil too, we should adopt strategies to evaluate the quality of professional medical practice, similar to the assessments conducted by the Brazilian Bar Association. Implementing such measures is crucial, considering the limited federal budget, which falls short of meeting the needs of the majority who rely on the public health system (SUS), and cannot afford wastage.

Teaching hospitals play an essential role in this regard, as they provide a platform for critically assessing innovative techniques, and ensuring new technologies are adopted responsibly and effectively.

## FOUNDATION OF PREVENTIVE MEDICINE: HEALTHY LIFESTYLE

When translating medical knowledge into practical applications for the general population, emphasizing the concept of a healthy lifestyle is paramount, particularly within the context of preventive medicine.

Most cardiovascular events, including myocardial infarction and death, are associated with modifiable risk factors such as dyslipidemia, smoking, hypertension, and diabetes.^5^ Genetic factors play a minor role in most cases. The Whitehall Study, conducted in England,^4^ demonstrated that public sector workers in lower hierarchical positions had a three to four times higher mortality rate than those in higher positions, further highlighting the influence of lifestyle on health outcomes.

The foundation of preventive medicine lies in adopting a healthy lifestyle, encompassing a diet rich in vegetables, fruits, and fish, coupled with reduced consumption of red meats and carbohydrates. Regular aerobic and strength exercises, at least 150 min/week, are strongly recommended, including for the protection of cognitive functions and the prevention of Alzheimer’s disease.^5^

Exercise and diet are crucial for preventing and treating diabetes, hypertension, and obesity. Numerous smoking cessation programs are available with considerable success rates. In the book “The Blue Zones,”^10^ American researchers examined the lifestyle of the five longest-living populations globally: Okinawa (Japan), Sardinia (Italy), Ikaria (Greece), Loma Linda (California), and Nicoya (Costa Rica). Common practices include a diet rich in grains, fruits, vegetables, and fish, with minimal red meat; a vibrant social life; spirituality; a strong emphasis on family; regular physical labor such as walking, tending to animals, cooking, and housekeeping; and limited use of medications. Genetic factors do not seem to be the sole explanation for this longevity, as the populations are from different countries with no familial relation.

Emotional stress from any source is a well-established causal factor in cardiovascular events. The exponential increase in such conditions during the coronavirus disease pandemic confirms this association.^11,12^

A unique challenge in promoting a healthy lifestyle lies in its implementation in adults, posing a significant obstacle for the third component of translational medicine: the general population. For instance, outcomes from initiatives to instill healthy habits in children and adolescents, as evidenced in Brazil and other countries,^13,14^ are striking—with children monitoring their parents to ensure they avoid smoking, exercise, and maintain a healthy diet. Hulsegge *et al*.^15^ found that individuals who sustained four to five healthy habits over 5 years had a 2.5-fold reduction in the risk of cardiovascular diseases and overall mortality compared to those who did not maintain these habits.

It is crucial to consider the setting in which such implementation takes place, whether in hospitals, educational programs, within the SUS, in private medical practice, during online consultations, or elsewhere. Different strategies are required depending on the context.

## TEAMWORK

Given the complexity of certain cases, comorbidities, varying institutional capabilities, and individual experiences, working in multidisciplinary teams is an effective strategy to provide comprehensive patient care. In the field of cardiology, a typical team should include a clinician, an interventionalist, a surgeon, or an electrophysiologist.^16^

In practice, the recommendation of procedures is influenced by individual experience. For instance, hemodynamicists may favor percutaneous interventions, whereas surgeons might prefer surgical procedures. There are arguments supporting either procedure based on its non-invasive nature, longitudinal outcome data, the efficacy of pharmacological treatments, and patient lifestyle considerations. Furthermore, the swift advancement of research techniques and treatments, along with the unique expertise of physicians and medical centers, may also lead to variations in opinions. Therefore, the heart team aims to reduce these biases. In these circumstances, it is essential to ensure that the patient is informed and consulted regarding their preferences.

## ESSENCE OF THE TRANSLATIONAL PROCESS: HIGH-QUALITY RESEARCH

The preceding arguments underscore the fundamental importance of scientific rigor throughout the translational medicine process. From the meticulous collection of experimental data in vitro*,* ex vivo*,* or in vivo*,* through the rigorous design and execution of clinical studies ranging from Phase I to III, to the responsible application of knowledge in the general population, scientific integrity must be paramount. Ideally, randomized clinical trials with well-defined, clinically relevant outcomes, adequate patient numbers, and appropriate follow-up duration are the preferred study design. However, implementing randomized trials is often hindered by substantial costs and delays in obtaining results.

Several factors clearly impact the translation of best practices into healthcare delivery for the population, including the off-label use of drugs, economic considerations, and misconceptions about exercising medical autonomy. Nevertheless, contemporary methods such as Mendelian randomization, Genome-Wide Association Studies, and big data analytics, enhanced by artificial intelligence, computational advances, and novel statistical techniques such as propensity score analysis, enable more comprehensive investigations that shed light on underlying causes and pathophysiological mechanisms.^17-19^

In the realm of interventions, clinical efficacy remains the primary concern for physicians. Ultimately, the credibility of medicine rests firmly on the principles of the scientific method.
